# Modeling long-term nutritional behaviors using deep homeostatic reinforcement learning

**DOI:** 10.1093/pnasnexus/pgae540

**Published:** 2024-11-28

**Authors:** Naoto Yoshida, Etsushi Arikawa, Hoshinori Kanazawa, Yasuo Kuniyoshi

**Affiliations:** Graduate School of Information Science and Technology, The University of Tokyo, Tokyo 113-8656, Japan; Graduate School of Informatics, Kyoto University, Kyoto 606-8501, Japan; Graduate School of Information Science and Technology, The University of Tokyo, Tokyo 113-8656, Japan; Graduate School of Information Science and Technology, The University of Tokyo, Tokyo 113-8656, Japan; Next Generation Artificial Intelligence Research Center, The University of Tokyo, Tokyo 113-8656, Japan; Graduate School of Information Science and Technology, The University of Tokyo, Tokyo 113-8656, Japan; Next Generation Artificial Intelligence Research Center, The University of Tokyo, Tokyo 113-8656, Japan

**Keywords:** homeostasis, deep reinforcement learning, nutritional geometry framework, nutrition

## Abstract

The continual generation of behaviors that satisfy all conflicting demands that cannot be satisfied simultaneously, is a situation that is seen naturally in autonomous agents such as long-term operating household robots, and in animals in the natural world. Homeostatic reinforcement learning (homeostatic RL) is known as a bio-inspired framework that achieves such multiobjective control through behavioral optimization. Homeostatic RL achieves autonomous behavior optimization using only internal body information in complex environmental systems, including continuous motor control. However, it is still unknown whether the resulting behaviors actually have the similar long-term properties as real animals. To clarify this issue, this study focuses on the balancing of multiple nutrients in animal foraging as a situation in which such multiobjective control is achieved in animals in the natural world. We then focus on the nutritional geometry framework, which can quantitatively handle the long-term characteristics of foraging strategies for multiple nutrients in nutritional biology, and construct a similar verification environment to show experimentally that homeostatic RL agents exhibit long-term foraging characteristics seen in animals in nature. Furthermore, numerical simulation results show that the long-term foraging characteristics of the agent can be controlled by changing the weighting for the agent’s multiobjective motivation. These results show that the long-term behavioral characteristics of homeostatic RL agents that perform behavioral emergence at the motor control level can be predicted and designed based on the internal dynamics of the body and the weighting of motivation, which change in real time.

**Significance Statement**

This study offers valuable insights into the long-term behavioral characteristics of homeostatic reinforcement learning (homeostatic RL) agents, showing that they can mimic the long-term foraging strategies of animals in nature. By applying the nutritional geometry framework from nutritional biology, we demonstrate that the multiobjective control of homeostatic RL can be adjusted to display various long-term foraging behaviors observed in animals. These findings enhance our understanding of bio-inspired autonomous systems and their potential applications in developing long-term operating household robots and agents for ecological simulations.

## Introduction

The ability to meet all of the different demands made on the body to survive in the environment is one of the sophisticated forms of intelligence seen in animals (1, 2). This problem can be reduced to the question of how to solve a multiobjective problem using a single body, such as how to satisfy all of the different conflicting demands (charging, temperature control, housework, replacing parts, etc.) (3). This situation also naturally appears in household robots and agents in complex recent computer games (4–6).

Solving such problems is an important issue for the long-term operation, learning, and development of autonomous agents, including robots. Is it possible to construct such sophisticated intelligence in autonomous agents, including their long-term characteristics?

In recent computational neuroscience, this type of problem setting has once again come into focus from the perspective of classical homeostasis, as homeostatic reinforcement learning theory (homeostatic RL) (7–9), based on discussions of interoceptive sensation in neuroscience (10–12). Homeostatic RL is composed of a combination of the concept of homeostasis, which states that the internal state of the body is maintained around a constant in physiology (13–15), and computational reinforcement learning theory (RL), which has a solid mathematical foundation in control theory and statistical machine learning (16, 17). Homeostatic RL constitutes a theory of adaptive behavior learning in which homeostasis serves as the sole motivational factor for learning.

By combining homeostatic RL with deep RL, a powerful behavioral optimization technique in recent years (18–21), various engineering insights have been obtained. This has shown that it is possible to emerge various behaviors at the motor control level, such as walking, navigation, and foraging control that depends on the internal state of the body, through motivation using only information from within the body (22–24). Furthermore, these behaviors are integrated and emerge from the perspective of the agent’s survival, with homeostasis as the sole objective.

However, although these agents apparently exhibit motor control and integrated behavior similar to that seen in animals, many aspects of their long-term behavioral characteristics are unknown, such as how similar they are to actual animals and how such long-term behavioral characteristics can be controlled.

Focusing on foraging in animals, obtaining food greatly contributes to the survival of animals, as well as their development, reproduction, health, and biological fitness (25). Due to this importance, animals’ foraging behavior has been modeled and studied at various time scales, from moment-to-moment motivation to long-term behavior (1, 2, 26).

In particular, the nutritional geometry framework (NGF) in nutritional biology is known as a powerful framework that can discuss the balance of multiple nutrient intakes in animal foraging behavior by measuring the total long-term intake of each nutrient, and various quantitative findings have been obtained on the long-term characteristics of foraging and their consequences in various animals (2, 27).

**Fig. 1.**
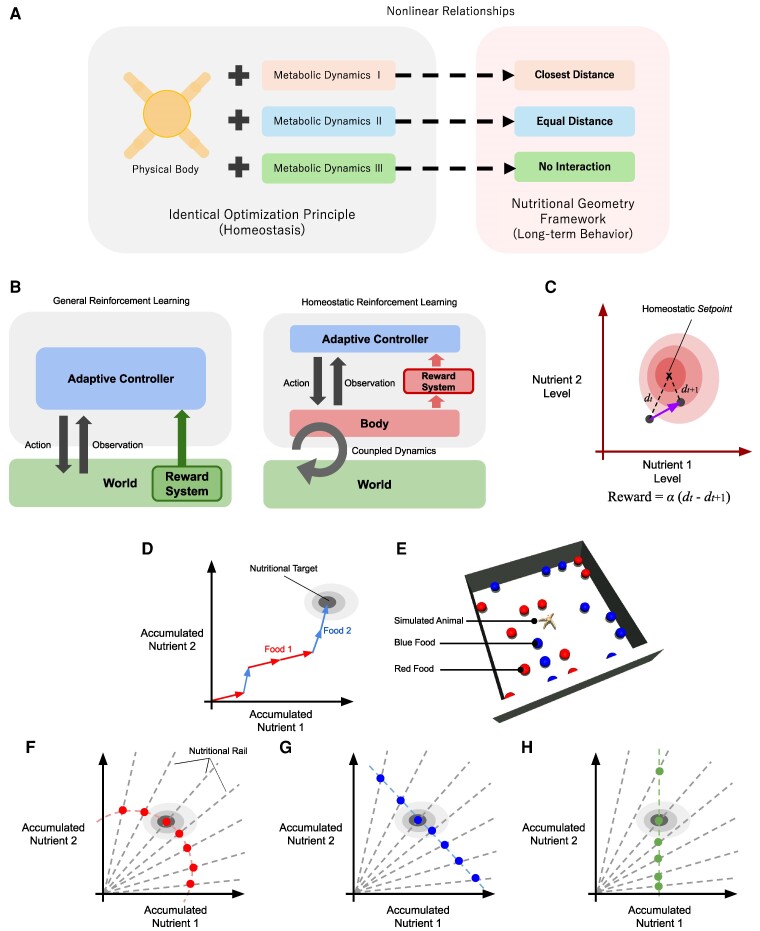
A) The modeling approach of our computational experiment. Animals in the simulation have physical bodies and have identical optimization principles for their internal nutritional state. The three different long-term nutritional behavioral types reported in the NGF studies can be expressed as differences in the body’s internal metabolic mechanisms and the behavior optimization. B) The structural differences between general RL (left) and homeostasis RL (right). In the concept of the learning agent in general RL (gray panel), the reward is assumed to be on the environment side, and the existence of the agent’s body is not explicit. In homeostasis RL, the existence of the autonomous agent’s body and the existence of a reward system on the agent’s side are explicitly assumed, and the homeostasis of the internal state of the body defines the reward. C) The state-space model of homeostatic RL. The 2D space represents the internal bodily state of the animal, which changes by metabolic dynamics and interactions between the animal and its environment. Reward signals for training are calculated using the temporal difference of distances $R=\alpha (d_{t}-d_{t+1})$, where *d* is the distance between the current nutritional state and homeostatic *setpoint* with a constant α>0. D) Nutritional space of NGF, where an animal consumes two types of macronutrients, represented by red and blue food ad libitum. The total amount of nutrients ingested changes as animals consume food. This means that the agent’s nutritional state shifts in the nutrient space (Food 1 and Food 2 arrows). Following a predefined period, the animal approaches the common *intake target* (IT) in the nutritional space (top right circle). E) Experimental conditions. Each animal has a 2D nutritional state. F–H) Three characteristic curves in NGF analysis (F: CD, closest distance; G: ED, equal distance; H: NI, no interaction).

In this study, we will investigate the long-term behavioral characteristics of homeostasis RL agents, which have been unclear until now, as well as how these are influenced by the physiological conditions of the agents, based on the analysis methods and findings on the long-term characteristics of real animal foraging behavior known in NGF researches. The concept of our modeling approach is illustrated in Fig. 1A. The contributions of this article can be summarized as follows.

- From the perspective of NGF with respect to foraging behavior, we construct a method for quantitatively evaluating the long-term behavioral characteristics of homeostasis RL agents that generate behaviors at the motor control level.
- We show that the characteristic curves corresponding to typical long-term behavioral characteristics reported in NGF can be modeled in simulations as differences in the metabolic dynamics of the agent’s body in homeostasis RL.
- Numerical simulations are used to clarify how parametric weighting of motivational factors corresponds to long-term foraging characteristics in NGF analysis, in multiobjective optimization in homeostasis RL.

The structure of this study is as follows. First, in Background section, we will explain the concepts of homeostasis RL and NGF in nutritional biology, which form the background to this study. In the Experimental settings section, we explain the details of the numerical simulations conducted in this study. In the Results and discussion section, we present the results of the simulations and discuss the results. In the Related works section, we describe the relationship between this study and previous studies on NGF, long-term behavioral characteristics of agents, and homeostatic RL. Finally, the Conclusion section summarizes the research.

## Background

### Homeostatic reinforcement learning

RL and homeostasis provide a bottom-up short-term explanation of behaviors.

Computational RL is a branch of machine learning that is an optimization-based method that calculates the rules (policies) for determining actions that maximize the total sum of future rewards based on the experiences gained by artificial agents through their interactions with the environment (including the dynamics within the body) (17). In RL, we assume that agent receives the current observation $x_{t}$ at time step *t* and takes an action $a_{t}$. And then, the agent receives the next observation $x_{t+1}$ and the temporal evaluation of the transition R(t) which is called *reward*. RL algorithms maximize the expected value of the following objective.


$$\sum_{t=0}^{\infty }\gamma^{t}R(t),$$

where γ∈[0,1) is the discount factor of the future rewards. Thus, rather than maximizing the most recent reward, the RL agent’s behavior is optimized to take the action that maximizes the sum of rewards over the future.

Homeostasis defines the way animals control their key internal states within a narrow range to maintain their bodily functionality (13, 15). The classical motivational theory suggests that homeostasis is the origin of motivational behavior (14, 28). Recent computational studies in neuroscience have reported that homeostatic RL (8, 9), along with homeostatic regulation and RL theories, can be used to understand adaptive behaviors using a normative reward definition.

Homeostasis RL is a subfield that deals with specific problem settings in RL, and places greater emphasis on the embodiment of agents. In contrast to general RL, homeostatic RL assumes that the reward given to the agent is constructed within the agent’s body. This is clearly shown in Fig. 1B. In general RL, the reward is set arbitrarily and is assumed to be an unknown function for the agent (Fig. 1B left). In this case, the rich dynamics that should be inherent in the actual animal’s body due to its interaction with the outside world are completely ignored. On the other hand, in the case of homeostasis RL, the reward is defined inside the agent’s body, and it is considered to be composed only of information within the agent (Fig. 1B right). This approach to rewards is a more natural and appropriate setting for assuming autonomous learning in animals (29–31).

In homeostatic RL, to achieve homeostasis through the autonomous learning of behavior by the agent, an internal target state (homeostatic *setpoint*) is assumed for the internal state of the body, and the homeostasis reward is defined using the temporal difference of the error between target and current internal states $d_{t}$ (Fig. 1C) (8, 9). Yoshida et al. (23) have reported that this definition of reward actually achieves homeostasis in the agent and has computational advantages.

Moreover, recent advances in deep RL (18–21) have enabled the direct behavioral optimization of simulated animals for homeostasis, including continuous motor control (22, 23, 32).

### Nutritional geometry framework

Nutritional biological studies have quantitatively and experimentally examined long-term foraging behaviors of various animals (26, 33). The NGF paradigm describes the balance between multiple nutrients (2, 27) assuming the consumption of multiple macronutrient types ad libitum: protein-rich and carbohydrate-rich, for example. After a certain period, animals typically maintain a balance between both nutrients by controlling their food consumption. The total amount of nutrients consumed is called the *intake target* (IT) (27) (Fig. 1D).

In addition, previous studies based on NGF have reported the results of measuring the nutrient intake of individual animals by feeding each animal a single type of feed with varying nutrient balances. In this case, if there is an imbalance in the nutritional composition of the food provided, the animal will suffer from conflicting appetites for different nutrients. As a result, this will lead to either an overeating or deficit of one nutrient, or a situation somewhere in between (26). In NGF, the pattern of the response to the nutritional imbalance is called the “rule of compromise” (26, 27, 34) and is evaluated based on actual animal experiments or observational data across various nutritional compositions (Fig. 1F–H): closest distance (CD), equal distance (ED), and no interaction (NI) (2, 27). These CD, NI, and ED are three examples of how different animals resolve this trade-off.

Points on the CD curve are located close to the IT on the nutritional rails (Fig. 1F). For the ED curve, the absolute difference in the amount of each nutrient type from the IT is equal (Fig. 1G). The NI curve represents a special case, where an arbitrary difference from the IT is permissible for one nutrient (Fig. 1H). Several studies suggest that the differences in responses to food imbalance may have an ecological origin related to differences in environmental conditions and access to food resources for insects (35, 36), and this is also discussed for primates (37).

### Modeling long-term nutritional behaviors by motor control

We modeled long-term nutritional behaviors of animals from the perspective of short-term continuous motor control. In our dynamic environment, we constructed a simulated quadruped animal model that includes metabolic dynamics (Fig. 1E). Subsequently, we adopted the model-free deep RL algorithm (19) along with the homeostatic reward setting for continuous motor control to optimize the animal. We set identical hyper-parameters for deep RL, including the reward function (8), while adopting different metabolic dynamics or motivational weights for each long-term behavior.

## Experimental settings

**Fig. 2.**
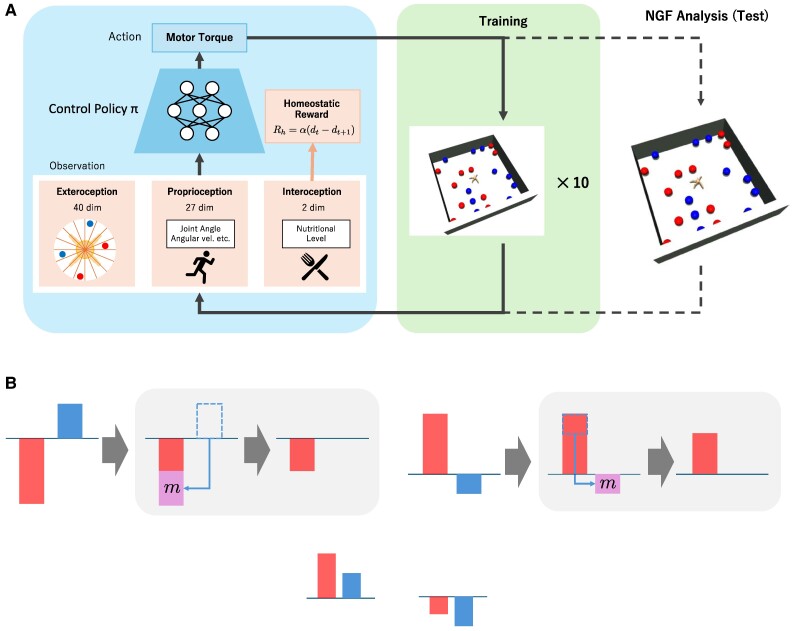
A) Training conditions for simulation animals. The torque of the joint motors of the simulation animal is calculated by a neural network (control policy *π*) with exteroception, proprioception, and interoception as inputs. The simulation animal is trained with rewards aimed at homeostasis of the internal state of the body, performed in a parallel training environment. The parameters of the optimized control policy are then fixed, and the behavior is evaluated in a test environment based on NGF analysis. B) Examples of metabolic dynamics in the ED rule. The left side of the top panels shows after the update of Eq. 1, while the center and right sides (gray background) show the update of Eq. 2 and its result. The two examples in the bottom row are examples where no change occurs after updating Eq. 2. If both nutrients are sufficient or insufficient, the update will not cause any change.

We established an experimental environment utilizing the MuJoCo dynamics simulator (38, 39). For our experiments, we employed a quadruped robotic agent with eight joints for control, a configuration inherited from a prior deep RL investigation (40). The configuration of the system in this study is illustrated in the Fig. 2A, where the control policy *π* is optimized by deep RL. The movements of these joints were controlled by 8D torque outputs (action, *a*) generated by the agent’s policy network, which is in a component of the actor-critic architecture in RL (19). Each decision step occurred at 0.05 [s] time intervals. The agent was free to explore its environment, which was enclosed by four walls.

The simulated animals had two types of nutritional states ($s_{\mathrm{blue}}$ and $s_{\mathrm{red}}$), which was represented as a 2D nutritional state. Within the scope of this study’s simulations, this nutritional state was assumed to be entirely hypothetical, rather than reflecting actual specific nutritional substances (carbohydrates, salt, protein, etc.) in the animals.

The environment contained ten units of each type of food balls. The colors of the food balls indicated a 2D internal nutritional state. When the agent’s torso was close to the center of a food ball, the agent consumed the food. As described in the next subsection, the nutritional state changes with predefined dynamics that are influenced by food consumption.

The agent’s sensory inputs (observation, *x*) consisted of a 27D proprioceptive input, 40D exteroceptive input, and 2D interoceptive input.

Proprioceptive inputs included the height of the agent’s torso from the floor, joint angles, joint angular velocity, torso orientation, and posture.

Exteroceptive inputs referred to the agent’s perceptual signals outside the body, consisting of egocentric rangefinder stimuli for two types of food balls. A range finder has a 20D output that gives normalized distances to the corresponding food type in ±180 degrees. Each dimension of the vector has a nonoverlapping angular range with a width of 18 degrees and then gives the normalized distance to the nearest food ball of the corresponding type. For example, the normalized distance in *k*th angular range for red type food is given by $1-\min(d_{k},d_{\max})/d_{\max}$, where $d_{\max}=5$ [m] is the maximum detection range and $d_{k}$ is the Euclidean distance in xy-plane between the center of the agent’s torso and the nearest red ball in *k*th angular range.

The interoceptive inputs were tied to the agent’s internal nutritional state, denoted as $s=[s_{\mathrm{blue}},s_{\mathrm{red}}{]}^{⊤}$. An episode concluded if the agent’s internal state s exceeded the unit hypercube boundaries of $[-1,1{]}^{2}$ (death) or if 60,000 time steps had elapsed. At the start of each episode, the agent’s internal state was initialized randomly, following a uniform distribution over the range of $[-1/6,1/6{]}^{2}$.

### Simulated metabolic dynamics

The metabolic dynamics in our computational models were defined, inspired by classical studies on the computational model of foraging behaviors (41, 42). In CD, both nutrients decrease at a fixed rate $\delta_{\mathrm{default}}$ over time, and a predetermined amount of nutritional resources were filled when the animal consumed food. Specifically, in our experiment we used a simplified metabolic dynamics


$$\begin{array}{rl} s_{\mathrm{red}}(t+1) & =s_{\mathrm{red}}(t)+\delta_{\mathrm{red}}^{c}(t),\\ s_{\mathrm{blue}}(t+1) & =s_{\mathrm{blue}}(t)+\delta_{\mathrm{blue}}^{c}(t). \end{array}$$

Where *t* is the time tick, and c∈{CD,NI}. The update rules of CD is


$$\begin{array}{rl} \delta_{\mathrm{red}}^{\mathrm{CD}} & =-\delta_{\mathrm{default}}+a_{\mathrm{red}}I_{\mathrm{red}}(t)+b_{\mathrm{red}}I_{\mathrm{blue}}(t),\\ \delta_{\mathrm{blue}}^{\mathrm{CD}} & =-\delta_{\mathrm{default}}+a_{\mathrm{blue}}I_{\mathrm{red}}(t)+b_{\mathrm{blue}}I_{\mathrm{blue}}(t), \end{array}$$

where $a_{\mathrm{red}}=b_{\mathrm{blue}}=0.1$, $a_{\mathrm{blue}}=b_{\mathrm{red}}=0$, and $\delta_{\mathrm{default}}=0.00015$. $I_{\mathrm{red}}(t)$ and $I_{\mathrm{red}}(t)$ are event functions that take 1 only when the simulated animal has consumed a food of the corresponding color, otherwise 0.

In NI, one nutrient remains constant and is instantly discarded upon consumption of the corresponding nutritional food. This operation is mathematically equivalent to ignoring the deviation of the corresponding nutrient from the setpoint. The update of NI is represented as


$$\begin{array}{rl} \delta_{\mathrm{red}}^{\mathrm{NI}} & =0,\\ \delta_{\mathrm{blue}}^{\mathrm{NI}} & =-\delta_{\mathrm{default}}+a_{\mathrm{blue}}I_{\mathrm{red}}(t)+b_{\mathrm{blue}}I_{\mathrm{blue}}(t). \end{array}$$

In contrast, in the metabolic dynamics of ED, we hypothesized a nutrient substitution mechanism in which any nutrient deficit is replaced by another nutrient that is already in excess. This is a simplified version of the gluconeogenesis-like process in real animals, assumed in the body of the simulated animal. In our model, ED uses two-step metabolic dynamics. The first steps is same with CD rule


(1) $${\tilde{s}}_{\mathrm{red}}(t)=s_{\mathrm{red}}(t)+\delta_{\mathrm{red}}^{\mathrm{CD}}(t),$$

and the ED rule uses the following modification step:


(2) $$s_{\mathrm{red}}(t+1)={\tilde{s}}_{\mathrm{red}}(t)+\Delta_{\mathrm{red}},$$

where


$$\Delta_{\mathrm{red}}=\left\{ \begin{array}{ccc} m, & \text{if}\;{\tilde{s}}_{\mathrm{red}}(t)<0\;\; & \;0>{\tilde{s}}_{\mathrm{blue}}(t)\\ -m, & \text{if}\;{\tilde{s}}_{\mathrm{red}}(t)>0\;\; & \;0<{\tilde{s}}_{\mathrm{blue}}(t)\\ 0, & \text{otherwise} \end{array}\right.$$

and


$$m=\min(|{\tilde{s}}_{\mathrm{red}}(t)|,|{\tilde{s}}_{\mathrm{blue}}(t)|).$$

The update of blue nutrition is defined similarly. The meaning of the second step is simple; If there is excess in a nutrition and deficit in a nutrition, the excess of the nutrition compensate the lack of other nutrition ($\Delta_{\mathrm{red}},\Delta_{\mathrm{blue}}$). See Fig. 2B for a visual description of the ED update.

The substitution mechanism of nutritions in terms of NGF framework was originally hypothesized by Simpson et al. (43), and later Cheng et al. (33) from the view point of the curve fitting of the NGF models to the biological data. In our simulations, their hypothetical substitution mechanism is replaced by a mechanism that is updated in real time.

### Reward definition

The reward functions are identical in all experiments and is composed of two components


$$R(t)=R_{h}-C,$$

where the first term is the homeostatic reward term (9), and *C* is the additional cost term to facilitate the training. The homeostatic term is the temporal difference of the distance from the setpoint


$$R_{h}=\alpha (d_{t}-d_{t+1}).$$

We used α=100 and *d* is the squared Euclidean distance between the current nutritional state s and the homeostatic target ($[0,0{]}^{⊤}$ in experiments)


$$d={s_{\mathrm{blue}}}^{2}+{s_{\mathrm{red}}}^{2}.$$

For the second term, we used the cost term


$$C=k_{p}\|p_{t}-p^{*}{\|}^{2}+k_{u}\|u_{t}{\|}^{2},$$

where the first term is the postural cost of the simulated animal. $p_{t}$ and $p^{*}$ are the actual posture of animal’s torso (roll and pitch) at timestep *t*, and the desired posture. In our experiment, we used the upright posture of the animal for $p^{*}$. The second term in the right side of the equation is the cost for the action $u_{t}$ (torque outputs). We used $k_{p}=0.005$ and $k_{u}=0.0005$ following our preliminary study (32).

### Behavior optimization

To optimize the behavior of simulated animals with given metabolic dynamics, we used an on-policy model-free deep RL algorithm PPO for the continuous motor control (19). We adopted the Python implementation of PPO in PFRL (44) as our codebase. We constructed the both value network and the policy network in the actor-critic architecture using the multilayer perceptron (45). The value network had 4-layer fully connected network (hiddens size: 256-64) with hyperbolic tangent activation function (tanh) and a single continuous output. The policy network had 4-layer fully connected network with tanh activation (hidden size: 256-64), and the output of the network consist of 8D Beta distribution (46).

For each experimental condition, we conducted 500 PPO iterations and observed convergence. As for PPO iteration, we collected rollout samples for training through 10 parallel sampler threads up to $3\times 10^{5}$ samples. The rollout samples were separated as minibatch with $5\times 10^{4}$ samples. For each iteration, 30 stochastic-gradient update epochs were conducted. Also, we set the clipping parameter of PPO at 0.3. We adopted the default setting in PFRL for all other hyper-parameters.

### NGF analysis of the simulated animals

The optimized agents were assessed under NGF conditions, all within the same physical environments as the training session, and used different food conditions. We systematically varied the nutrient conditions using nutritional rails. In this testing session, the parameters in simulated animals were fixed and no training updates were conducted.

To follow the NGF experiment conducted to real animals, we tested the simulated animal in the following condition for 8,000 time steps. In the test condition, the numbers of food resource are same (red:blue = 10:10); however, nutritional contents were identical for all food resources; $a_{\mathrm{red}}=b_{\mathrm{red}}=0.1\beta$ and $a_{\mathrm{blue}}=b_{\mathrm{blue}}=0.1(1-\beta )$. We tested the optimized simulated animal with conditions from β=0.2 to 0.8, respectively. We ran 20 sessions for each *β*. We used only the results of the agents that survived 8,000 steps to compute the statistics.

We observed the amount of total nutrient consumed by agent, for each red and blue nutrition. The results of the amount should on the nutritional rail (y=xtanβ) (27). The standard deviations in the plot were calculated along with the rails.

### Long-term behavioral control based on drive weighting

From the perspective of data fitting based on NGF, Cheng et al. (33) discussed how NI could be treated as the limit of CD by considering what kind of minimization of the metric is applied to the points on nutritional rails from IT. Specifically, by assuming weighting for the error between IT and each nutritional intake, the difference between the two long-term behavioral characteristics (NI and CD) can be expressed as a kind of spectrum depending on the difference in weighting (33). In the limit where an infinite weight is assumed for a single type of nutritional axis, NI can be well expressed.

Inspired by this discussion, in our simulation based on microscopic time scales, we used a metabolic simulation based on the CD rule and compared the results by weighting drives for each nutrient. We investigated the possibility that different long-term behavioral characteristics (CD, NI, and their spectrum) could be controlled in the simulated animals, depending on the differences in the weighting of individual drives.

To do so, we evaluated how weighting of the drives for the blue and red nutritional states contributed to the agents’ long-term behavior. Concretely, we introduced a weighted drive function according to the following equation and compared its impact on the NGF analysis results by training agents using various weightings of the drives with rewards based on this function


$$d=\left(\frac{2w_{\mathrm{red}}}{w_{\mathrm{blue}}+w_{\mathrm{red}}}\right){s_{\mathrm{red}}}^{2}+\left(\frac{2w_{\mathrm{blue}}}{w_{\mathrm{blue}}+w_{\mathrm{red}}}\right){s_{\mathrm{blue}}}^{2},$$

where the weights $w_{\mathrm{blue}}$ and $w_{\mathrm{red}}$ are positive constants, respectively, specifying the relative intensity for the individual drives corresponding to each nutrient. This weighting implies


$$\frac{2w_{\mathrm{blue}}}{w_{\mathrm{blue}}+w_{\mathrm{red}}}+\frac{2w_{\mathrm{red}}}{w_{\mathrm{blue}}+w_{\mathrm{red}}}=2$$

and


$$\frac{2w_{\mathrm{blue}}}{w_{\mathrm{blue}}+w_{\mathrm{red}}}/\frac{2w_{\mathrm{red}}}{w_{\mathrm{blue}}+w_{\mathrm{red}}}=\frac{w_{\mathrm{blue}}}{w_{\mathrm{red}}}.$$

Also $d={s_{\mathrm{blue}}}^{2}+{s_{\mathrm{red}}}^{2}$ when $w_{\mathrm{blue}}=w_{\mathrm{red}}=1$. This is equivalent to the drive function defined earlier.

In the experiment, we optimized the agents as in the previous experiment by varying the weights for blue nutrients as $w_{\mathrm{blue}}\;:\;w_{\mathrm{red}}=1\;:\;1$, 2:1, 4:1, 8:1, and 16:1, respectively, and performed NGF analysis on each agent.

We evaluated the 10,000-step time averages of the red and blue interoceptive errors (${s_{\mathrm{blue}}}^{2}$ and ${s_{\mathrm{red}}}^{2}$) in the same environment as the training environment for the obtained agents and compared them (N=100).

Then, we measured the long-term characteristics of the agents by running the NGF analysis on each agent. In this case, 20 NGF analyses were performed on each agent with different random number seeds. Since a sufficient number of agents could not survive 8,000 steps depending on the angle of the nutritional rail, the average was calculated only for results with more than 10 surviving agents and used for comparison.

## Results and discussion

**Fig. 3.**
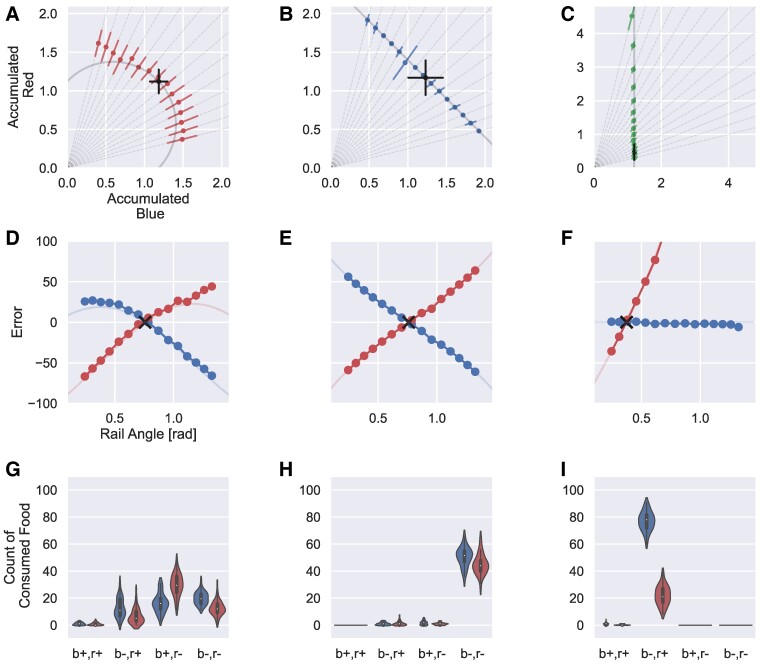
A–C) Results of NGF analysis of simulated animals optimized with three metabolic dynamics (n=20 for each rail, A: CD, B: ED, C: NI). Broken lines represent expected curves (27). Black points denote the expected IT obtained by deploying the optimized animal in the training field. The locations and variances are different because IT is evaluated for each condition. D–F) Evaluation of errors from IT. The horizontal axes measure the angles of nutritional rails, whereas the vertical axes represent the error from the IT ($\frac{v-t}{t}\times 100$, where *v* is the angular value and *t* is the target). The curves with dots represent the average accumulated nutrients, while thin lines represent the error between the theoretical and measured results. Crosses represent the corresponding IT (D: CD, E: ED, and F: NI). G–I) Count of the first 100 consumed food resources under each condition (n=50, G: CD, H: ED, and I: NI). The horizontal axes represent the internal state conditions of the agent immediately before food capture (x+ means $s_{x}>0$, where x∈{red,blue}, and vice versa).

Figure 3 shows the emergent long-term foraging behaviors under each condition. Figure 3A–C depicts the total consumption of the simulated animals under the test conditions. The black dots represent the total intake of each nutrient obtained by allowing the optimized simulated animals to forage freely in the training environment under each condition, which corresponds to IT.

The characteristic curves obtained from the three computational experiments capture three distinctive long-term behaviors in the NGF, which have been previously observed in locusts (Fig. 1F–H). Figure 1B exhibits a small variance for some nutritional rails. We observed that the ED agent approached the food nearly deterministically and periodically before stopping, which may explain this variance.

Figure 3D–F depicts error curves from the NGF perspective, which correlate with the theoretical outcomes from the calculated ITs (27). In the CD setting, we observed excessive errors in the high-angle region of the red nutrient compared to the theoretical values. This may be caused by the incongruent conditions between the training and testing sessions. If the animals faced a deficit in the blue nutrient under the CD condition, they tended to collect blue food balls owing to their experience from the training session. However, the test session primarily included red nutrients, with a small amount of blue nutrients. Consequently, the animals continued to collect blue food balls while experiencing an excess of red nutrients.

Figure 3G–I presents the distributions of the first 100 food balls captured during training at specific internal state conditions. These distributions allow us to observe more detailed behavioral tendencies of the simulated animals. Under the CD strategy, animals exhibited selective food choices to satisfy their nutritional deficiencies. By contrast, animals under the ED strategy consumed both foods only when they were deficient in both nutrients.

### Drive weighting interpolates CD and NI

**Fig. 4.**
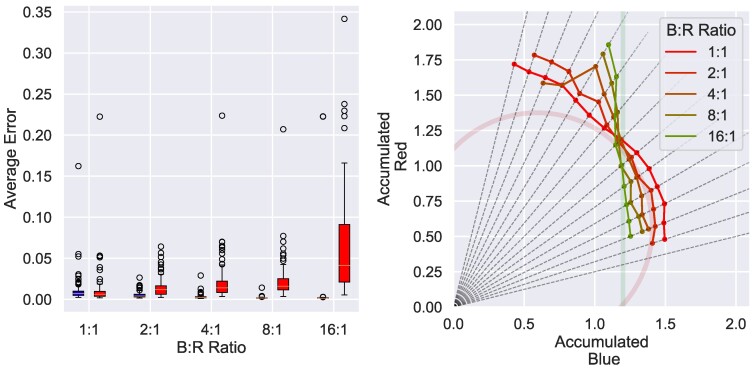
Results of the evaluation of agents optimized under the metabolic conditions of CD, with different weightings of the drives for each nutrient. Left: Boxplot of time-averaged interoceptive errors for each nutrient over 10,000 steps (N=100). The horizontal axis represents the ratio of weightings for nutrients, and the vertical axis represents the amount of error. Right: results of 20 NGF analyses for each drive weighting condition. However, the average of each result is calculated only for the surviving conditions of the 20 trials, and the results for conditions with less than 10 survivals are omitted. The circle represents the ideal CD condition, and the thick vertical line represents the ideal NI condition (parameters are the same as A and C in Fig. 3).

The left panel of Fig. 4 summarizes the results of the weighting for each component of the drive (blue and red). The left panel compares the time averages of the interoceptive errors obtained according to the weight of each drive. As expected, it illustrates that the interoceptive error for the red nutrient was relatively increased due to the greater weight given to the blue nutrient.

The right panel of Fig. 4 plots the results of the NGF analysis overlaid according to the ratio of each drive. The curves indicated by the thick lines are the ideal curves for CD (red) and NI (green), respectively, and are the same as those shown in Fig. 3A and C.

These plots show that the long-term behavior was similar to the CD behavior when the ratio was 1:1, but as the bias increased (red→green), it approached the NI behavior. This means that NI can be interpreted as a special case of CD, as explained in the description of the metabolic dynamics of NI. By reducing the relative weighting to the interoceptive error for red nutrients, the optimization allows for over- and under-intake of red nutrients. Thus, as is in the macroscopic modeling in NGF (33), NI can be understood as a result of the limit $w_{\mathrm{blue}}/w_{\mathrm{red}}\to \infty$ in terms of homeostatic drives.

The results of our modeling, which compute the behavior of simulated animals including the emergence of basic motor skills, show that it is possible to express various long-term characteristic curves seen in animals in nature by changing the parameters of the weighting on internal drives of the simulated animals.

## Related works

NGF-based simulations have been studied previously by Lihoreau et al. and Senior et al. (47, 48). Their models were agent-based simulations of animal populations, and they assumed long-term behavior of the CD type in advance. To handle the interaction between individuals and groups, they greatly simplified the dynamics of the environment. Their simulation was an abstract model that only dealt with the nutritional space of each individual, and it omitted the physical interaction between the individual agent and the environment. They used an evolutionary algorithm to optimize the agents using a fitness function defined by the error between the IT and the nutritional space and evaluated the behavior of the resulting simulated animals.

Similarly, Lihoreau et al. (49) investigated the impact of food distribution on the collective behavior of a group of agents by optimizing a population of agents assuming CD with an evolutionary algorithm . They also treated lifespan-fecundity trade-offs using a similar approach (50). These previous NGF-based approaches assumed CD, basic motor skills of the agent, etc. in advance and discussed the behavioral emergence resulting from explicitly incorporating the agent’s nutritional state into the fitness function. Both of these approaches are based on the existence of CD and do not discuss how CD, ED, and NI arise from more basic components.

As for more direct prior studies based on our methods, Yoshida and Kuniyoshi (51) have attempted to reproduce CD at the motor control level by training simulated animals with deep homeostatic RL in a setting similar to the present study, including visual input. Our study is an extension of this study to explain all of the long-term behaviors reported by (27) using the homeostatic RL modeling.

A different perspective from NGF, bottom-up modeling of animals’ long-term behavioral characteristics based on optimization principles was conducted by Wispinski et al. with deep RL (52). They modeled patch selection in optimal foraging theory using simulated animals (53). Their approach used a simplified animal model with predefined basic motor elements such as locomotion and then performs behavioral optimization using deep RL to discuss behavioral properties in the resulting simulated animals (52).

In our simulations, the reward is defined based on the homeostasis of multidimensional internal states inside the body. On the other hand, their approach assumes a single type of food and introduces a decreasing reward along the time spent in each patch (52). Recent computational neuroscience is debating the origin of rewards in RL models for animals (8, 31), and our reward setting based on homeostatic RL would provide more natural condition (31).

Explanations of animal behavior based on control theory have been made in classical studies (1, 54); however, they have included conceptual difficulties such as motivation and drive, as well as measurement difficulties (33). In overcoming these difficulties, NGF is a theory that deals with foraging behavior and the control of nutrition in animals, and it has an explicit measurement target of total nutrient intake, and it can deal with the intake of nutrients, behavior, and their consequences in many types of animals, including insects, birds, and mammals, including humans (26, 33, 36, 55–58). On the other hand, there has been a lot of research in the neural computation theory of animal behavior and learning based on control theory, including rodents such as mice, based on computational RL, which has a strong mathematical foundation (59–63). Our simulation study can be interpreted as a revisiting of the long-term behavior of simulated animals based on modern control theory and homeostasis, from the perspective of NGF.

## Limitations

We were able to model the long-term behavior of animals by optimizing the behavior of the agent based on homeostasis. Compared to real animals, the model used in this study has several limitations.

Firstly, the metabolic model used in this study is a very simple mathematical model based on the classical homeostatic RL literature (41), and while it is effective for discussing hypothetical metabolic dynamics, it may be too far from realistic settings. Therefore, to discuss more realistic overall animal models, more detailed digestive models are needed. For this, it might be possible to utilize previous research on modeling that expresses the actual digestive tract of animals as a chemical reactor (64–66). Furthermore, the metabolism of actual animals has complex dependencies, such that the depletion of specific nutrients becomes a bottleneck for the utilization and metabolism of other nutrients (67). Dynamical systems with complex dynamics have memory effects of their own, and there is potential for them to be utilized in learning (68).There is discussion about the relationship between the dynamics of insect hemolymph and learning (67). By discussing homeostatic RL as an abstracted learning algorithm that incorporates the complex dynamical systems within the body, it may be possible to view metabolism from the perspective of information processing.

The second is the adaptability of the agent. Animals change their behavior according to the rules of the situation through learning about food. Several studies has reported that it is possible to condition insects to respond to olfactory (69) and visual cues (70) in order to solve the depletion of specific nutrients in the body. These conditions are formed through exposure to training environments for several hours. This adaptation to new situations may be modeled as a process in which agents are pretrained in motor control and foraging behavior in a common general environment, and then a single agent is exposed to a new situation and fine-tuned by online behavioral optimization based on homeostatic RL.

In addition, Gradd and Raubenheimer (69) reported a result in which conditioning was observed for protein but not for carbohydrates in the conditioning of insects to odor stimuli and food containing specific nutrients. This suggests that nutrient-dependent learning occurs in response to the depletion of specific nutrients, and this is beyond the scope of this study. It is an interesting question how biased learning, which is not uniform across all nutrients, arises and how it is modeled in homeostatic RL.

## Conclusion

NGF and homeostatic RL are two theories that explain nutritional balancing behavior. NGF provides a rigorous approach to the relationships between nutrition, lifespan, and reproduction, whereas homeostatic RL describes behaviors and adaptations at the level of continuous motor control. Our simple computational model connects the two theories using the internal metabolic dynamics of animals.

Based on these results, our method can be applied in a variety of contexts. First, it can be used effectively in behavioral simulation research related to animal fitness, especially at the level of continuous motor control. In addition, when ecological simulations using a large number of agents are expected, it may be better to introduce the agents as autonomous learning agents rather than designing each agent manually. In such cases, the method of constructing the learning agents in this study allows for the design of agents that automatically acquire specific behaviors in the environment after specifying long-term characteristics of behaviors for each individual. Furthermore, as an engineering application, our research would enable us to predict what long-term characteristics will be derived from how drives and metabolic dynamics are provided in autonomous robots or simulation agents that assume long-term learning based on homeostasis.

## Data Availability

All data and code from our study are available at https://github.com/ugo-nama-kun/deeprl_gfn.
